# A Case Report of Long COVID or Post-COVID-19 Symptoms and Characteristics

**DOI:** 10.7759/cureus.63876

**Published:** 2024-07-05

**Authors:** Marika Mdivnishvili, Darejan Mdinaradze, Ketino Virkovi, George Gogashvili

**Affiliations:** 1 Cardiac, Thoracic, and Vascular Surgery, Jerarsi Hospital, Tbilisi, GEO; 2 Internal Medicine, Simon Khechinashvili University Clinic, Tbilisi, GEO; 3 Medicine, Caucasus's International University, Tbilisi, GEO; 4 Emergency Department, Simon Khechinashvili University Clinic, Tbilisi, GEO

**Keywords:** covid-19 prevention, sars-cov-2 and covid-19, long covid syndrome treatment, long-covid syndrome, long-covid-19

## Abstract

The coronavirus disease 2019 (COVID-19) caused by the severe acute respiratory syndrome coronavirus 2 (SARS-CoV-2) has led to a global health crisis. Long COVID refers to a debilitating condition characterized by severe symptoms that may arise after the initial acute phase of COVID-19. Significant attention has been directed toward the acute phase of the respiratory system while overshadowing the understanding and management of long-term complications, often referred to as "long COVID."

This case focuses on a 19-year-old female who experienced the multisystemic manifestation of COVID-19 syndrome several months after the initial infection, spanning cardiovascular, respiratory, endocrine, central nervous system, and multi-skeletal domains. This study aims to describe the patient's experience and recovery process with a specific emphasis on the long COVID experience.

## Introduction

The worldwide spread of coronavirus disease 2019 (COVID-19) has led to severe respiratory disease outbreaks. Globally, 770 million people have been diagnosed and around 7 million are reported dead [1]. Despite the health system's strive to stop the spread of SARS-CoV-2, long-term COVID-19 recognition has been limited and gone unnoticed within the healthcare systems, particularly in our environment. Termed as long COVID, chronic COVID, and long hauler COVID, post-COVID-19 syndrome was defined by the World Health Organization (WHO) [2]. This condition was observed when active cases came down and the patient recovered, but new symptoms arose, or the development of symptoms extended beyond 12 weeks [3]. The National Institute for Health and Care Excellence (NICE) provided guidance that encompassed signs and symptoms of the acute and persistent phases of COVID-19. This included having acute symptoms for a period of four to 12 weeks, referred to as ongoing symptomatic COVID-19, and for 12 weeks or longer, referred to as post-COVID-19 syndrome [4]. Presentation blurred the lines between acute and chronic illness and over time extended beyond physical health, imposing social and psychological burdens. Recent research findings underscored the value of understanding and evaluating COVID-19 ramifications because 70% of the population exhibited at least one organ impairment, particularly among those who were perceived as less vulnerable to severe outcomes [5].

## Case presentation

On 07/10/2023, a 19-year-old female presented to the hospital with worsening chest pain, abdominal discomfort, low-grade fever, shortness of breath, non-productive cough, and general weakness, which escalated approximately one week ago. A few hours before hospitalization, the patient had severe breathing difficulties at home and exhibited signs of respiratory failure, orthopnea, tachypnea, and oliguria, which is why she was taken to the hospital by ambulance brigade. She had pre-existing conditions such as chest and abdominal pain persisting for several months. The patient was initially diagnosed with gastritis but symptoms continued and was unresponsive to treatment. The patient also reported experiencing low-grade intermittent fever (37.8°C) for several weeks.

During her stay at the hospital, she remained conscious and responsive. Vital signs were notable for a heart rate of 146 bpm, blood pressure of 120/78 mmHg, respiratory rate of 26 breaths per minute, and oxygen saturation of 91% on room air.

A physical exam showed swelling of the genitals, which prompted consultation with a gynecologist. A systemic connective tissue disorder was suspected, and consultations with a rheumatologist and infectious disease specialist were sought. However, blood tests for antinuclear antibodies (ANA) and antineutrophil cytoplasmic antibodies (ANCA) returned negative results.

On 02/02/2023, the patient initially presented with mild respiratory symptoms during her acute COVID-19 illness, which resolved without requiring special treatment. However, a few months later, she developed an array of debilitating symptoms affecting multiple organ systems, including respiratory, cardiovascular, nervous, endocrine, digestive, urinary, genital, and musculoskeletal systems. These symptoms persisted and significantly impacted her daily functioning and quality of life. The patient was vaccinated only one time on 02/03/2022. She also noted insomnia, exertional dyspnea, and fatigability since May 2023. Special medical attention was given to the joint pain, which arose in June 2023, and received treatment with undisclosed medications. When collecting the medical history, it became clear that the patient had amenorrhea for six months.

**Figure 1:**
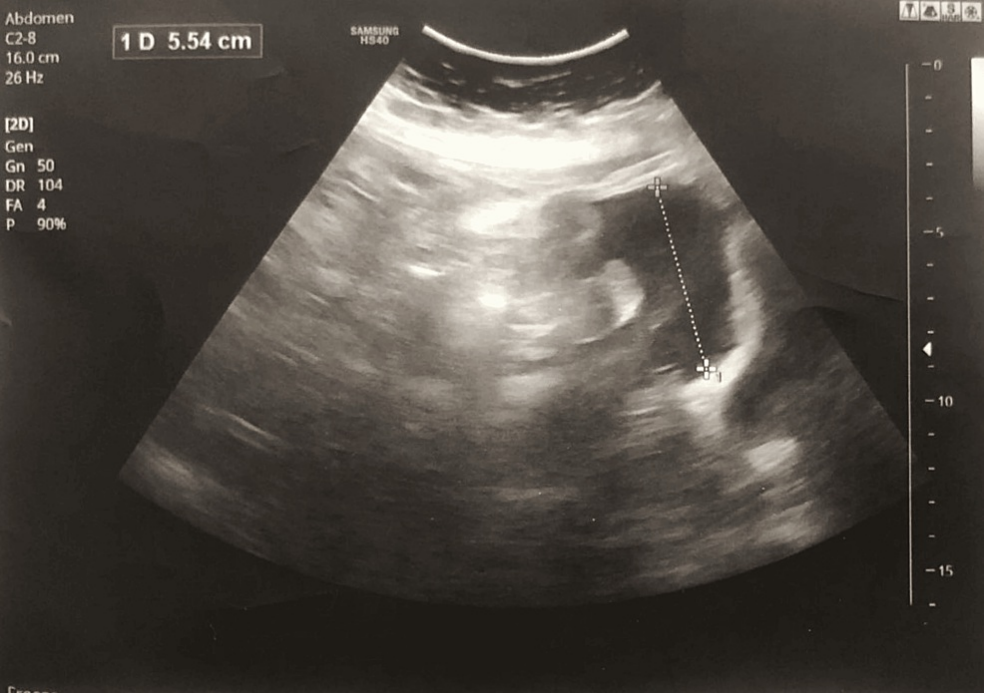
Abdominal ultrasonography

**Figure 2:**
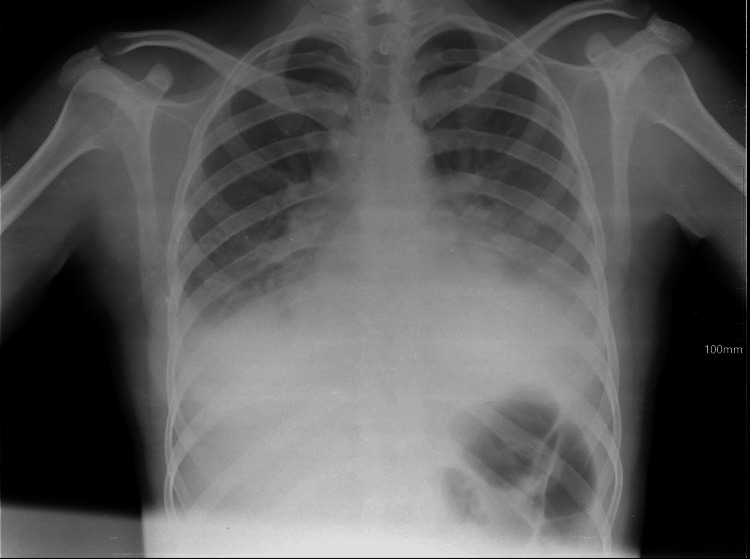
Patient's pleural effusion and extensive consolidation

A cardiologist, general surgeon, and gynecologist examined the patient. Clinical laboratory investigations of chest X-ray, CT of the chest cavity, ultrasonography, electrocardiogram (ECG) of the chest cavity, CT of the abdomen, and ultrasonography of the organs of the abdominal cavity results were essentially normal (Figure 1), except for bilaterally decreased breath sounds. Chest X-ray showed signs of free-flowing sterile pleural effusion (Figure 2). Gram stain and culture were negative. Ultrasonography of the pleural cavity showed free fluid in the right pleural cavity, an average of 800 ml, and an average of 700 ml of free fluid in the left pleura. On CT of the chest and abdomen-bilateral hydrothorax, lung fields were bilateral, and middle and lower fields showed extensive intensive consolidation.

The patient was admitted to the intensive care unit (ICU) due to the severity of her condition. The patient urgently received a multidisciplinary treatment approach (Table 1). This included continuous oxygen therapy, gastroprotective agents, antibacterial therapy, bronchodilators, antiarrhythmics, anticoagulants, and symptomatic management. Urgent thoracocentesis was performed, resulting in significant relief and improvement in respiratory distress. Subsequent monitoring revealed oxygen saturation levels of 97-98% with continuous oxygen therapy. Additionally, the patient was detected to have iron deficiency anemia, and iron preparation was added to the prescription.

**Table 1:** Medical treatment

Medical treatment	
Solution NaCl 0.9%	500 ml intravenously, 75 ml/h
Capsule omeprazole	20 mg 2x p/o (by mouth)
Ratiocef 1 g + solution NaCl 0.9%	20 ml 2x intravenously
Tablet Azimac	500 mg 1x p/o (by mouth)
Solution Berodual	1.0 ml 2x inhalation
Solution Corsair	0.5 mg/ml 2ml
Tablet Coraxan	7.5 mg 2x p/o (by mouth)
Solution Fraxiparine	0.4 1x s/c (subcutaneous)
02 oxygen therapy	6-7 l/min

After admission to the ICU department on 07/10/2023, the treatment was given for four days with the mentioned drugs and the condition stabilized dramatically. The patient was discharged on 10/10/2023. Dexamethasone 4 mg and oxygenation therapy were prescribed.

Proper treatment and management were received, and her symptoms improved at the end of the week. All the major symptoms resolved at the end of the second week but low-grade recurrent fever, headache, nausea, and abdominal pain persisted. The constellation of symptoms, coupled with a recent history of COVID-19 infection, suggests a potential complication or sequelae of the viral illness. Further diagnostic evaluation and management are warranted to address the acute respiratory distress and multi-system involvement observed in this patient.

## Discussion

Understanding the factors that contribute to long COVID is crucial as it helps to prioritize at-risk populations and design effective interventions. Studies highlight that the most influential predictors of long COVID-19 are the severity of the initial COVID-19 illness and subsequent hospitalization admission.

Studies suggest that the virus can trigger immune responses leading to cytokine storm. Widespread inflammation throughout the body can cause long-lasting organ damage [6]. Logic also aligns with findings from a systematic review, which identified hospitalization during the acute phase as the primary predictor of post-COVID syndrome [7]. Experiencing more than five symptoms during the acute phase of COVID-19 significantly increases the risk of developing long COVID [8]. The symptoms identified as predictors were fatigue, myalgia headache, dyspnea, and hoarse voice. Fatigue is the most common among long COVID symptoms [9]. Our patient had more than two of these predictor factors.

Another important predictor for long COVID was the presence of pre-existing conditions like hypertension and diabetes. There are several articles indicating the correlation between prolonged viral shedding and the associated development of long COVID. The extended duration of viral shedding in certain cases further complicates the clinical picture. This association stresses the need for heightened awareness and active management within clinical settings. In individuals with prolonged shedding, infectiousness should be determined via viral culture due to possible transmission. Risk factors for prolonged viral shedding are immunosuppression and irrational use of corticosteroids in managing COVID-19 patients [10]. Our patient did not have any immunocompromising conditions or previously identified risk factors, so this highlights its imperative for further research.

Recent recognition indicates that vaccination against SARS-CoV-2 does not guarantee protection from long COVID. There are several studies suggesting that COVID-19 vaccination could improve long COVID symptoms [11]. In another study, some long COVID symptoms, such as hair loss and ocular issues, worsened after vaccination [12]. On the contrary, one study did not find an association between long COVID and vaccination [13]. It is crucial to address pre-existing risk factors such as diabetes, high blood pressure, and obesity following COVID-19. Moreover, these conditions have emerged as a notable predictor for long COVID-19. By identifying these predictors, clinicians can promote improved physical function through lifestyle changes, target prevention strategies, and smoking cessation efforts.

Several additional pathophysiological mechanisms have been proposed, including an altered angiotensin-converting enzyme 2 (ACE2) receptor expression, immune dysregulation, pulmonary and endothelial dysfunction, systematic inflammation, and hypercoagulation. There are various hypotheses to explain the link between new-onset diabetes and COVID-19 infection. One potential mechanism revolves around the idea that SARS-CoV-2 alters glucometabolic control by damaging the pancreas. ACE2 is a crucial payer in glucose within the pancreas, as it serves as a primary attachment and gateway for SARS-CoV-2 entry into the host cells [14]. It leads to pancreatic damage and causes diabetes by reprogramming cells to produce more glucagon and less insulin. Additionally, excess inflammation and immune cell hyperactivation contribute to insulin resistance and exacerbate the risk of diabetes [15]. SARS-CoV-2, by binding and damaging islets, diminishes the ability of the pancreas to release insulin in response to the resultant hyperglycemia.

A possible explanation for the development of new-onset hypertension following SARS-CoV-2 infection includes the disruption of the renin-angiotensin-aldosterone system (RAAS). Angiotensin II promotes blood vessels to narrow and raises blood pressure levels. In essence, the dysregulation caused by SARS-CoV-2 to the RAAS pathway raises angiotensin II levels and causes vasoconstriction, resulting in hypertension [16].

Lipids serve a vital function in regulating the immune system’s activity. SARS-CoV-2 can lead to a "cytokine storm" by inflammation throughout the body and disrupt the balance of lipids in the bloodstream, leading to a condition termed immune-mediated inflammatory dyslipoproteinemia. Specifically, lower levels of high-density lipoprotein referred to as “good’’ cholesterol impede its ability to neutralize pathogen-associated lipids, which are involved in mediating immune response, thereby contributing to prolonged inflammation [17].

Studies revealed a higher occurrence of long COVID among women because of variations in sex hormones, which contributed to differences in autoimmune response and influenced the pathogenesis of long COVID [18]. Hormones influence heightened inflammatory state during the acute phase, even after recovery [19]. Additionally, females have been noted to have stronger production of IgG antibodies during the early phase of the disease.

Our findings underscore the multifaceted nature of long COVID and highlight the importance of considering both the severity of the initial illness and underlying health conditions in predicting its occurrence.

## Conclusions

This article has presented a case of a 19-year-old female who developed symptoms (shortness of breath, generalized fatigue, tingling sensation, disturbed sleep, and feeling of apprehension) after recovery from COVID-19. All the possible causes were ruled out and the development of such symptoms even after viral clearance (after COVID-19 recovery) led to the conclusion of post-COVID syndrome. Post-COVID syndrome is a relatively new topic and its description is not uniform among various studies due to which there is no absolute definition of post-COVID syndrome. However, NICE guidelines state that post-COVID syndrome develops after an infection consistent with COVID-19, continues for more than 12 weeks, and is not explained by alternative diagnosis. Further investigations should be directed toward pathophysiology, risk factors, and management strategies for evolving conditions.
